# Age-Onset-Related Particularities of Pediatric MS—Understanding the Spectrum: A Tertiary Center Experience

**DOI:** 10.3390/diseases13070193

**Published:** 2025-06-25

**Authors:** Alice Denisa Dică, Dana Craiu, Florentina Ionela Linca, Magdalena Budișteanu, Catrinel Iliescu, Carmen Sandu, Cristina Pomeran, Diana Bârcă, Niculina Butoianu, Carmen Burloiu, Ioana Minciu, Ina Ofelia Focșa, Dana Surlică, Oana Tarța-Arsene, Cristina Cazacu, Andreea Badea, Alexandru Stefan Niculae, Daniela Adriana Ion

**Affiliations:** 1 Pediatric Neurology Department, Prof. Dr. Alex. Obregia Clinical Hospital of Psychiatry, 041914 Bucharest, Romania; alice.dica@drd.umfcd.ro (A.D.D.); catrinel.iliescu@umfcd.ro (C.I.); carmen.sandu@umfcd.ro (C.S.); cristina.pomeran@umfcd.ro (C.P.); diana.barca@umfcd.ro (D.B.); niculina.butoianu@umfcd.ro (N.B.); carmenburloiu@gmail.com (C.B.); ioana.minciu@umfcd.ro (I.M.); dsurlica@gmail.com (D.S.); maria-cristina.calusaru@drd.umfcd.ro (C.C.); andreea.badea@umfcd.ro (A.B.); nalexandru.stefan@yahoo.com (A.S.N.); 2 Pediatric Neurology Department, University of Medicine and Pharmacy Carol Davila, 020021 Bucharest, Romania; daniela.ion@umfcd.ro; 3 Department of Special Psychopedagogy, Faculty of Psychology and Educational Sciences, University of Bucharest, 0506578 Bucharest, Romania; 4 Psychiatry Research Laboratory, Prof. Dr. Alex. Obregia Clinical Hospital of Psychiatry, 041914 Bucharest, Romania; laborator.cercetare.psihiatrie@spital-obregia.ro (M.B.); ina.focsa@umfcd.ro (I.O.F.); 5 Department of Genetics, Faculty of Medicine, Titu Maiorescu University, 031593 Bucharest, Romania; 6 Medical Genetics Laboratory, Victor Babes National Institute of Pathology, 050096 Bucharest, Romania; 7 Department of Medical Genetics, Faculty of Medicine, University of Medicine and Pharmacy Carol Davila, 020021 Bucharest, Romania; 8 Department of Pathophysiology, National Institute for Infectious Diseases Prof. Dr. Matei Balș, 021105 12 Bucharest, Romania

**Keywords:** challenge, children, early onset, particularities, multiple sclerosis

## Abstract

Background: Pediatric-onset multiple sclerosis (POMS) is a rare and heterogeneous condition, with clinical features, progression, and therapeutic response varying significantly according to age at onset. Early-onset MS (<10 years) presents particular diagnostic and management challenges due to atypical presentations and more active inflammatory profiles. Objectives: To identify age-related clinical, radiological, and therapeutic characteristics of pediatric MS, with a specific focus on early-onset cases, and to compare them with intermediate (10–12 years) and late-onset (>12 years) forms. Methods: We conducted a retrospective analysis of medical records from 120 pediatric patients diagnosed with MS at a tertiary neurology center between 2018 and 2024. Patients were grouped by age at onset and assessed for clinical presentation, number and timing of relapses, EDSS scores, imaging findings, and treatment patterns. Results: Early-onset MS was associated with atypical symptoms, delayed diagnosis, more frequent relapses, and multifocal brainstem and cerebellar involvement. The diagnosis was significantly delayed in younger children compared to adolescents. EDSS scores tended to remain stable in the first 2–3 years, but early-onset patients showed a notable decline after the fourth year. While most patients received disease-modifying therapies, high-efficacy agents were underused due to age-related restrictions. Intermediate-onset patients presented overlapping features of both early and late-onset MS and had the highest proportion of fully preserved motor function (EDSS 0) at the end of follow-up. MRI findings revealed more extensive and confluent lesions in younger patients, particularly in the first two years after onset. Conclusions: Age at disease onset is a key determinant of clinical course and treatment response in pediatric MS. Early recognition and timely initiation of appropriate therapy—especially high-efficacy agents—may improve outcomes and reduce long-term disability. Further multicenter studies with standardized imaging and cognitive assessment protocols are needed to optimize care for this vulnerable population.

## 1. Introduction

A neurodegenerative, immune-mediated disease affecting the brain and spine, multiple sclerosis (MS) is common in young adults and rare in pediatric age [1,2,3,4,5,6,7]. Between 3% and 5% of all MS cases start at <18 years, with early-onset pediatric MS affecting very few children, <1% [8,9,10,11,12,13].

Recently, early-onset multiple sclerosis (EOMS), usually defined as beginning before age 10, has been the focus of researchers and doctors in contrast to late-onset multiple sclerosis (LOMS) (onset between ages 10/11/12 and 18) due to distinct patterns in clinical presentation, disease progression, cognitive involvement, and long-term outcomes [14,15,16,17,18,19,20,21,22]. Thus, until now, the comparison was between two groups: early (before 10–12 years) and late (after 10–12 years) onset pediatric MS or between children and adults (this aspect was not discussed in this paper). Also, there are few publications about the early onset group (before 10 years), especially from Eastern Europe.

In EOMS, the typical female predominance observed in MS is often absent [23,24,25]. Clinical features in pre-pubertal children are usually multifocal, frequently involving the brainstem and cerebellum. Presentations may include ataxia, cranial nerve palsies, acute disseminated encephalomyelitis (ADEM)-like episodes, or acute motor deficits. In contrast, late-onset cases tend to be mono-focal at onset, commonly presenting with optic neuritis, sensory disturbances, or unilateral motor weakness [17,19,23,24,25,26,27,28,29,30,31,32].

POMS has more relapses than adult MS [6,8,11,12], particularly in early-onset [16]. In this age group, also more than in late-onset ones, in the first 2–6 years, high disease activity was observed [17,33], but in most of them, the recovery is complete in the first episodes; that’s why the deficit accumulation and EDSS score modification are slower [1,9,33,34,35]. Over time, motor and cognitive impairments are similar to those of adults or worse [8,10,36,37]. Thus, MS at a pediatric age developed a relapsing-remitting form in most patients rather fast from the beginning.

Cognition is affected from the beginning at a young age of onset [12,38] in at least one-third of the cases [39,40], first seen as a lack of concentration, information processing speed [9,34,41,42] problems, and eventually a drop in IQ. Psychiatric concerns in late childhood onset are first more clear, shown by behavioral disorders and sadness; however, these problems also affect small children with MS, with a high impact on the quality of life of all POMS patients [26,43,44,45,46,47,48].

MRI shows more new and active lesions in children than in adults [14,15,16,17,18,19,20,49], and in very young ones, extensive lesions and atrophy are present from the beginning [23] owing to very active inflammatory processes, particularly in the first 2–5 years of the disease [50,51,52,53].

Oligoclonal bands are less present in pre-pubertal age than in adolescents [19,23,30]; therefore, the number of relapses and new lesions evident on imaging is often used to track dissemination over time [4,19].

Diagnosing MS in young children can be particularly challenging due to atypical clinical presentations and non-specific imaging findings, often leading to diagnostic delays and postponed initiation of disease-modifying therapies (DMTs) [35,54].

Talking about the treatment of pediatric multiple sclerosis, we still have few molecules available and even fewer with high efficacy [7,35], usually the latter being used off-label by pediatric neurologists for the treatment of active forms, pending the completion of ongoing clinical trials with highly efficient therapies [7,35,55,56,57,58].

Variability in clinical presentation and illness course according to the age of beginning within the juvenile spectrum further challenges experts, complicates management techniques and impacts the quality of life of patients and their families [59,60,61].

## 2. Materials and Methods

This retrospective study examined medical records of children with MS assessed and treated in our Pediatric Neurology department from January 2018 to December 2024, from which 120 individuals were chosen. According to the McDonald 2017 revised criteria, all patients were diagnosed with MS—clinically isolated syndrome (CIS) or recurrent remitting form (RR MS).

For each patient, we documented the clinical presentation at the initial demyelinating event, the number of relapses (attacks), and the interval between them.

Although non-specific, cerebrospinal fluid (CSF) markers such as oligoclonal bands and IgG index were considered important indicators of inflammatory activity.

Neuroimaging using 1.5T MRI (brain and spine), both native and with contrast enhancement, was performed at disease onset or during the first hospital admission. MRI scans were subsequently repeated at each relapse or at scheduled intervals—six months after onset, diagnosis, or initiation of treatment, and annually thereafter—in accordance with current clinical guidelines. Disease progression was assessed over time based on the presence of new and/or active lesions, both prior to and after the initiation of immunomodulatory therapy.

To evaluate neurological disability and disease progression before and after the start of disease-modifying therapy (DMT), we employed the Expanded Disability Status Scale (EDSS) [62], a standard tool in MS assessment.

Psychiatric and psychological evaluations were conducted to assess cognitive impairment, depression, anxiety, and behavioral disturbances associated with pediatric MS, and individualized therapeutic interventions were implemented accordingly.

Regarding the DMTs, most of the patients—9 children each from Groups 1 and 2 and 86 patients from Group 3—received IFNb or fingolimod, only three had RTX, and one with dimethyl fumarate abroud. A total of 6 and 7 patients from Groups 1 and 2, respectively, and 79 from Group 3 have only one DMT; all the others have 2 or 3, switching from IFN to Fingolimod/RTX or from IG to IFN and again to Fingolimod (Table 1).

Data analysis was performed using SPSS version 22 and JAMOVI version 0.17.1.0. Descriptive statistics (frequencies, percentages) were used to summarize clinical and demographic characteristics. Inferential analyses included chi-square tests to assess associations between categorical variables and the Kruskal–Wallis test to compare distributions across three age-related onset groups. To ensure consistency across groups, a random sample of ten patients with MS onset after the age of 12 was selected for subgroup analysis. Non-parametric tests were applied as appropriate due to data distribution characteristics.

Informed consent was obtained from all parents or legal guardians. The study protocol was approved by the Ethics Committee of the Clinical Hospital “Prof. Dr. Alexandru Obregia” (approval no. 8377/25 March 2025).

## 3. Results

### 3.1. Demographic and Clinical Characteristics

The study cohort consisted of 120 pediatric patients diagnosed with MS: 41 boys (34.17%) and 79 girls (65.83%) (Table 1).

At the time of the first demyelinating event, 11 patients were under 10 years of age (Group 1), 10 were between 10 and 12 years (Group 2), and 99 were older than 12 years (Group 3). MS diagnosis occurred at <10 years in 8 patients, between 10–12 years in 5, and after age 12 in 107. In most cases, the first attack coincided with the time of diagnosis (5 patients in Group 1, 6 in Group 2, and 99 in Group 3) (Table 1).

Sex ratios varied: Group 1 showed a male predominance (1:2 female to male), Group 2 had an equal distribution, and Group 3 had a female predominance (2:1) (Table 1).

**Figure 1:**
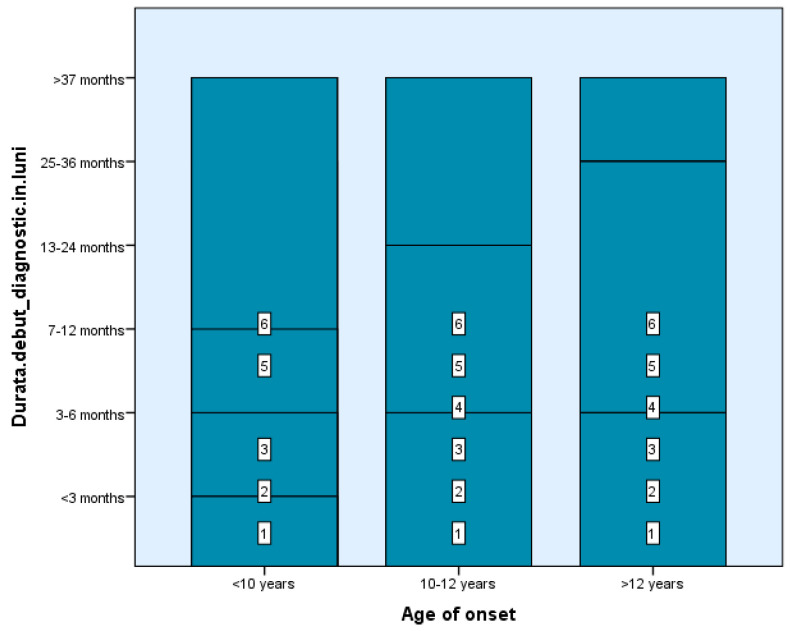
The time between the first demyelinating episode and the MS diagnosis.

Among our study groups, we found that the time between the first demyelinating episode and the MS diagnosis was fairly short—between 0 and 6 months—for four children in Group 1 and four subjects in Group 2. In contrast, 61 of the 99 teenagers got a diagnosis in one month (2–4 weeks) following the first event, the typical duration of investigation completion, which suggests the simplicity of diagnosis at an older age of onset where the features of the condition resemble those of adulthood (Figure 1). There are statistically significant differences between groups depending on the duration from the first demyelinating episode to receiving the diagnosis, Χ^2^(2) = 4.71, *p* < 0.05, *p* = 0.048 (Table 1 and Table 2).

Relapsing-remitting MS (RRMS) was the predominant form: 9 patients in Group 1, 6 in Group 2, and 62 in Group 3. The remaining 43 had clinically isolated syndrome (CIS) (Table 1).

### 3.2. Relapse Profile and Timing

In Group 1, three children experienced a single relapse before diagnosis, seven had two events, and one had three. Group 2 patients had between one (n = 7) and three (n = 3) relapses. Most Group 3 patients (65/99) were diagnosed after their first episode (Table 1).

Following diagnosis, four patients in Group 1 and four in Group 2 had additional relapses (1–4 and 1–2, respectively) before treatment. In contrast, 38 Group 3 patients had no post-diagnosis relapses prior to starting treatment. There are statistically significant differences between groups depending on the duration from the diagnosis to treatment, Χ^2^(10) = 25.46, *p* < 0.01, *p* = 0.005 (Table 1 and Table 2).

Total relapses ranged from 2–16 in Group 1 (5 had ≥5 events), 2–8 in Group 2 (5 had ≥3), and 2–4 in 54 patients from Group 3. The longest inter-episode interval (≥37 months) was observed in 3 patients from Group 1 and 2 from Group 2. Most late-onset patients (Group 3) had inter-relapse intervals of 0–12 months and up to 6 total episodes. There are statistically significant differences between groups depending on the total events, Χ^2^ (2) = 5.21, *p* < 0.05, *p* = 0.047 (Table 1 and Table 2).

Three Group 1 children and two Group 2 patients experienced more than 37 months between the first and second episodes, which was the longest time between assaults documented. By contrast, several Group 3 late-onset (43) individuals had a shorter time between the first two relapses, ranging from 0 to 12 months. Subjects from Groups 1 and 2 had more occurrences than those with a late start (Group 3) at the same time, with a shorter overall event duration (<12 months in most cases). Late-onset patients experienced a maximum of six debilitating episodes. There are statistically significant differences between groups depending on the duration between the first and second episodes, Χ^2^ (2) = 22.21, *p* < 0.05, *p* = 0.035 (Table 1 and Table 2).

### 3.3. Clinical Presentation at Onset

Early-onset patients (Group 1) frequently presented with brainstem and cerebellar involvement, including multiple cranial nerve deficits (n = 4), ADEM-like symptoms (n = 3), hemiparesis (n = 4), and bilateral optic neuritis (n = 1). Four had multifocal presentations.

Group 2 patients showed hemiparesis, vestibular/cerebellar symptoms, cranial nerve involvement, sensory disturbances, and ADEM-like presentations.

For juvenile patients with late-onset (Group 3), MS begins with hemiparesis (n = 35), paresthesias and sensitivity issues (n = 32), and optic neuritis (n = 28), vestibular syndrome (n = 17), occasionally linked with intracranial pressure syndrome (ICH) and chronic headache (n = 2).

### 3.4. Neurological Impairment During Relapses

To evaluate the neurological impairment during relapses, we considered the following items: unilateral motor deficit (one limb or one hemibody—hemiparesis, monoparesis), bilateral motor deficit (all limb or both LL/UL—bipyramidal syndrome, diparesis, tetraparesis), sensory disturbances (one or multiple locations, for all types of sensitivity, including paresthesia and associated pain), balance and gait disorders (cerebellar ataxia, vestibular syndrome, intentional tremor/trembling), cranial nerve involvement (one or multiple), optic neuritis (uni- or bilateral), ADEM-like (especially in very young patients)/intracranial hypertension syndrome (especially in Balo’s type lesions) (Table 1).

- Group 1 had four children with unilateral deficiency throughout three to five events;
- Group 3 had seven children with similar motor dysfunction during one event. In all research groups, bilateral deficiency is uncommon.
- While 35 late-onset individuals had between 1 and 4 sensory disturbance episodes, most Group 1 and 2 patients had 1–2 episodes.
- Of the early-onset MS kids, nearly half (5) experienced between 1 and 8 episodes of balance problems; 20 from Group 3 had 1–3 episodes. Of Group 3, 21 individuals have optic neuritis during 1 to 4 episodes, which is less common in the other two age-related groups.
- Cranial nerve involvement seems more common in Groups 1 and 3—1–3 attacks; in Group 2, however, four people suffer this impairment in 1 to 4 relapses.
- Early onset (three individuals) is more frequent for ADEM-like appearance, while intracranial hypertension syndrome is more prevalent in late-onset participants with tumefactive or prolonged lesions (Table 1).

Group 2’s individuals experienced hemiparesis, vestibular/cerebellar syndrome, many cranial nerve involvement, ADEM-like sensitivity problems, and paresthesias.

### 3.5. CSF and MRI Findings

Oligoclonal bands (OCBs) were present in 5 Group 1 patients (2 converted from negative at the second event), 8 Group 2 patients, and 81 from Group 3 (Table 1).

Initial brain MRIs revealed 5–10 lesions in 6 Group 1, 5 Group 2, and 64 Group 3 patients. Between 11–20 lesions were seen in 4 patients each from Groups 1 and 2 and 23 from Group 3. Active plaques (4–6) were found in 5 Group 1, 2 Group 2, and 16 Group 3 patients.

There were no statistically significant differences between the 3 groups in terms of the number of brain and spinal cord lesions at the first MRI, *p* > 0.05 (Table 1 and Table 2).

Lesion topography differed by age: younger patients had larger, confluent lesions in lower brain regions; adolescents had smaller, round, periventricular lesions typical of MS.

Most of the patients underwent a new MRI after 6 months—6 from Groups 1 and 2, and 60 from Group 3—that revealed 7 to 10 new lesions in three subjects and 1 to 6 demyelination in 4 children from Group 1. One to six lesions were found in six and forty patients from groups two and three, respectively (Table 1). Group 1 youngsters had more spine lesions on MRIs from disease onset and six months (Table 1).

New lesions were most frequent in the first 3 years and declined after treatment initiation, especially from year 3 onward (Table 1).

### 3.6. Therapies and Treatment Response

Disease-modifying therapies (DMTs) included interferons, fingolimod, rituximab (RTX), and, in one overseas case, dimethyl fumarate.

DMTs were administered to 9 patients each in Groups 1 and 2 and 86 in Group 3.

Most patients (6 in Group 1, 7 in Group 2, 79 in Group 3) received only one DMT; others switched treatments, usually from IFN to fingolimod or RTX.

Highly effective therapies (HETs) were used as first- or second-line agents in 4 Group 1, 6 Group 2, and 5 Group 3 patients.

All patients were followed for at least one year, with the majority monitored for two to three years. Younger patients had longer follow-ups, often exceeding five years.

**Figure 2:**
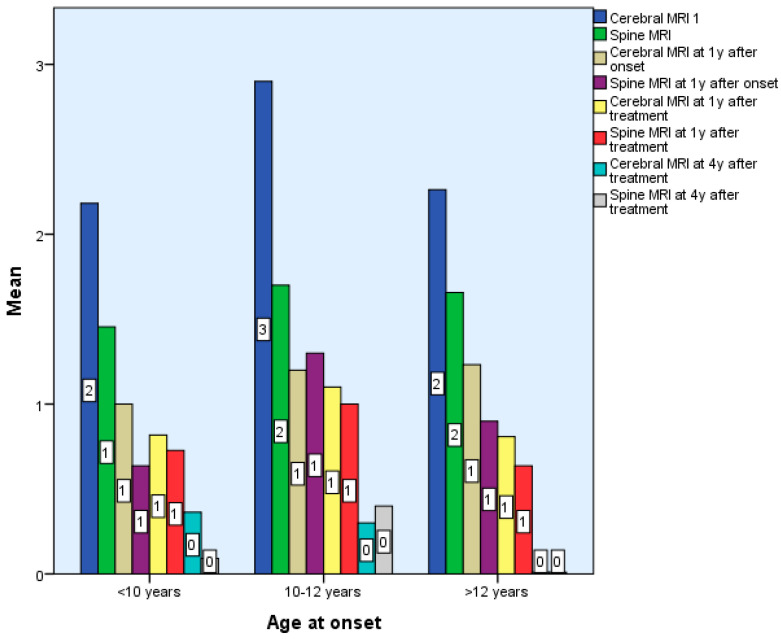
Differences according to age of onset of multiple sclerosis in terms of Cerebral and Spinal MRI.

Following treatment, the number of new lesions decreased, mostly from the third year of treatment. That is why we think the decrease in the number of new lesions is the reaction to therapy. There are no statistically significant differences between the 3 groups in terms of the number of brain and spinal cord lesions in the first 6 months after diagnosis *p* > 0.05, but there are statistically significant differences in terms of the number of spinal cord and brain lesions 7 years after treatment, *p* < 0.01 (Table 1 and Table 2, Figure 2).

### 3.7. Disability and Cognitive Outcomes

Considering the EDSS score in relation to the time of diagnosis after 6 months, two of the patients with early onset, nine with late-onset, and one from Group 2 exhibited modest motor skills impairment (EDSS 1-2), while one patient with late-onset was significantly impacted. Two of the Group 1 children, 2/5 of the roup 3 children, and one Group 2 child exhibited moderate motor impairment (EDSS 2.5–3) at 2–3 years post-diagnosis. Three early-onset kids exhibited moderate disability four, five, and six years following diagnosis. There are statistically significant differences between groups according to EDSS scores 4–7 years after diagnosis and treatment, *p* < 0.01. However, there were no statistically significant differences between EDSS scores between 6 months and 3 years after diagnosis and treatment, *p* > 0.05 (Table 1 and Table 2).

Patients with late and early onset who begin DMTs have superior functioning than preteens (2 patients with moderate concerns) after 3 years of particular treatment. Limited functionality after the treatment onset reveals a critical period that manifests in the first three years of treatment for three Group 3 patients with severe motor skills impairment (EDSS > 3) after one year of immunomodulatory therapy, followed by two subjects with severe impairment at two and three years of treatment (Table 1).

At final evaluation:

- Group 1: 6/11 had EDSS 0, 4 had mild impairment (EDSS 1–2), and 1 had severe disability.
- Group 2: 7/10 were fully functional (EDSS 0), 2 had mild, and 1 had severe impairment.
- Group 3: ~10% had EDSS 1–2; three had moderate, and five had severe impairment.

**Figure 3:**
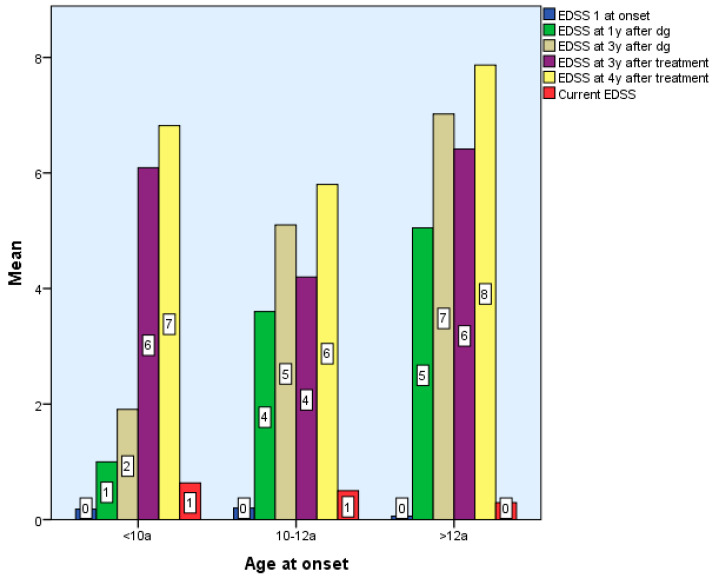
Differences according to age of onset of multiple sclerosis in terms of EDSS score.

There were no statistically significant differences between the EDSS scores obtained by the 3 groups at the end of the period, Χ^2^(6) = 9.34, *p* > 0.05, *p* = 0.155 (Table 1 and Table 2, Figure 3).

### 3.8. Neuropsychological Outcomes

Cognitive assessments revealed:

- Group 1: two children with borderline intelligence, two with mild intellectual disability, and others with executive and learning deficits.
- Group 2: four with borderline intelligence, two with MS-related anxiety and depression.
- Group 3: 4 with mild intellectual disability, 1 with severe cognitive impairment, and 30 with associated depression.

## 4. Discussion

The primary objective of this study was to explore the specific features of multiple sclerosis progression across different pediatric age groups. The 120 included patients were categorized by age at onset into three groups: early-onset (<10 years, Group 1), intermediate-onset (10–12 years, Group 2), and late-onset (>12 years, Group 3). Particular attention was given to the early-onset group, where we anticipated the greatest clinical and biological variability.

Like in the previous two groups, the gender distribution was balanced; in group three, a modest female sex preponderance (2:1) is seen, but less than in other research where the ratio is 3/4:1 [16,23,24,25].

Sex distribution was relatively balanced in Groups 1 and 2, while Group 3 showed a modest female predominance (2:1). This is lower than what has been reported in other studies, where the female-to-male ratio in pediatric MS typically ranges between 3:1 and 4:1 [9,13,14,15].

In children under 10 years and those aged 10–12, the interval between the first demyelinating event and confirmed MS diagnosis was notably longer—often exceeding six months. This delay was primarily due to atypical clinical presentations, such as ADEM-like episodes and incomplete diagnostic workups. In several cases, lumbar puncture was not performed at the beginning, which limited the identification of oligoclonal bands in cerebrospinal fluid and hindered the fulfillment of McDonald’s 2017 criteria. In contrast, most late-onset patients (aged > 12) were diagnosed within one month, typically following the completion of standard investigations. The time to diagnosis differed significantly between Groups 1 and 2 versus Group 3, indicating a statistically significant delay in younger age groups, *p* = 0.005, *p* < 0.01.

Most patients in our cohort, particularly those with early disease onset (80%), were diagnosed with the relapsing-remitting form of MS, regardless of age at onset. This may reflect a combination of diagnostic delay and the natural course of the disease, as also supported by previous studies [16,17,18,19].

Prior to diagnosis and initiation of disease-modifying therapies, all groups experienced multiple relapses. Notably, children in the early-onset group experienced approximately 2.5 times more attacks than those in the late-onset group, reflecting a more active inflammatory phase. While this difference was not statistically significant, it aligns with existing literature that describes higher relapse rates in younger patients [6,16,17,18,19,33]. A particular feature observed in our cohort was the longer interval between the first two demyelinating episodes in some early-onset cases, in certain instances extending to three years. This was followed by shorter intervals between subsequent relapses and a higher total number of episodes compared to older children. Unlike findings reported by Kuppke et al. and Kauth et al., where the time between initial attacks was similar across pre- and postpubertal groups, our data suggest a more heterogeneous pattern of disease evolution in younger children.

Clinically linking ADEM-like (27.27%), ataxia (36.36%), and numerous cranial nerve involvement (36.36%), the manifestations at the beginning (first event) and in the subsequent episodes indicate greater infratentorial lesions at a very early age. At an older age, the clinical features at the beginning and in evolution point to supratentorial involvement: hemiparesis (14.14%), sensitivity disorders (14.14%), optic neuritis (6.06%), and vestibular syndrome (23.23%), outcomes comparable but not exact—lower values on items than those reported [16,17,18,30].

Oligoclonal bands were positive in 63% of the subjects with early onset and in 80–85% of those from Groups 2 and 3 [19,23,30]; therefore, although inflammatory processes are very active at a young age, with the onset of adolescence, intrathecal production of inflammation increases. The positivity in our groups is similar to that described by Deiva et al. [23] as being reported between 50 and 90%.

Approximately 85% of patients across all age groups received disease-modifying therapies (DMTs). Among the remaining 15%, the reasons for not initiating treatment varied by age group. In Groups 1 and 2, some children did not meet the minimum age requirements for inclusion in the National Rare Disease Treatment Program, while others refused treatment due to the discomfort associated with frequent injections. In Group 3, a subset of patients who were approaching the age of 18 chose to delay treatment in order to become eligible for adult protocols and gain access to highly effective therapies (HETs) that are not yet approved for pediatric use.

In Group 1, 54% of children received low-efficacy DMTs, such as interferons (IFN), and 18% were treated with moderate-efficacy agents like fingolimod. One-third of the group required treatment escalation to fingolimod or rituximab (RTX), the latter administered off-label [7,35]. In Group 2, treatment was initially distributed equally: 40% received IFN, 40% fingolimod, and 10% RTX off-label. Over time, treatment adjustments led to a final distribution of 30% on IFN, 50% on fingolimod, and 20% on RTX—largely due to suboptimal response or adverse events. Among Group 3 patients, 81% were treated with IFN, 5% with fingolimod, and most are expected to transition to highly effective therapies (HETs) as they reach adulthood. Currently, access to HETs for pediatric MS remains limited in Romania, available only through clinical trials or off-label use, as there is no national program providing these treatments for pediatric-onset MS. This context explains the widespread use of an escalation approach in pediatric MS, despite evidence from the literature suggesting that early initiation of highly effective therapies is crucial in active forms of the disease [7,19,35,55,56].

Our findings differ somewhat from those recently released by Kauth, who reported in their study the use of low-efficacy treatment in 62.7% of EOMS and 55% of LOMS, moderate efficacy in 3.9% vs. 21.1%, and HET in 15.7% vs. 13.8%. Compared to our groups, in early and intermediate age, we used more moderate-efficacy treatment but a very high percentage of low-efficacy molecules for LOMS.

The Expanded Disability Status Scale (EDSS) remains a valuable tool for monitoring disease progression in pediatric MS, particularly with regard to motor function. In our cohort, we observed that EDSS scores tended to remain stable during the first 2–3 years following disease onset in all age groups (*p* > 0.05, *p* = 0.59–0.187), with more pronounced changes emerging from the fourth year onward—especially in children with early-onset MS (*p*< 0.001). With treatment, the EDSS score is slightly improved (*p* > 0.05, *p* = 0.052–0.584) or stationary in all three study groups (*p* < 0.001), which is statistically significant. Our findings support previous observations that functional recovery following early relapses is generally favorable in children, particularly after the first event, and that disability tends to accumulate slowly in the initial years. This is consistent with reports indicating delayed EDSS progression in pediatric populations [1,12,13,14,15].

Treatment with interferon beta (moderate efficacy) and fingolimod as high efficacy DMT have contributed to the stabilization or modest improvement of EDSS scores across all age groups, suggesting that early therapeutic intervention may help slow the accumulation of motor deficits, even when other new high-efficacy agents—monoclonal antibodies (natalizumab, ocrelizumab, alemtuzumab, etc.) are not available [9,16,33,34,35]. At the end of the study (7 years of monitoring), in Group 1, EDSS was zero in 54% and mildly affected (1-2) in 36%, with one at EDSS 5; in Group 2, 70% had EDSS 0 and 20% had EDSS 1-2, with one at EDSS 3; in Group 3, 84% had EDSS 0 and 10% had EDSS 1-2, with five at EDSS 3. The results for early and intermediate-age groups resembled those of the Kauth study, where the EDSS score at the end of the monitoring period was 93.5% [16].

Initial MRI scans revealed a progressive increase in lesion load from Group 1 to Group 3. In very young patients, lesions were more extensive, confluent, and often associated with perilesional edema, while adolescents displayed lesion patterns more typical of adult-onset MS in both appearance and location. New lesions were most frequently observed during the first one to two years after disease onset, particularly in early-onset patients. Following the initiation of disease-modifying therapy, a clear radiological improvement was seen, with lesion size reduction and absence of new or active plaques—findings that align with previous studies [16,17,18,19,23].

Despite treatment, approximately 20–30% of patients in Groups 1 and 2 required therapy escalation due to disease progression, as indicated by new relapses and radiological activity. These results underscore the need for close MRI monitoring in the early years of disease and support the potential benefit of more aggressive therapeutic strategies in select cases.

All kids with early onset age exhibit cognitive and behavioral issues; 20% had liminal intelligence and another 20% minor mental impairment, but they all have compromised executive function [38,39,40,41,42]. In contrast, psychiatric symptoms such as anxiety and depression were more prevalent in adolescents (Group 3), affecting at least half of the patients. These findings are consistent with previous reports highlighting the emotional burden of MS during adolescence [44,45]. In the intermediate-onset group (ages 10–12), cognitive and psychiatric manifestations frequently coexisted, underscoring the complex and transitional neurodevelopmental stage of preadolescence and the need for multidisciplinary support.

In summary, the early-onset MS group exhibited the most distinct clinical profile within our cohort. These patients were characterized by atypical presentations, more frequent and severe relapses, accelerated disease progression, and earlier onset of both motor and cognitive deficits. These features not only complicate diagnosis and management but also demand heightened clinical vigilance and tailored therapeutic strategies.

The intermediate-onset group (ages 10–12) appeared to represent a transitional stage between early childhood and adolescence in the clinical course of MS. These patients exhibited a combination of features observed in both the early- and late-onset groups in terms of clinical manifestations, MRI findings, and treatment response. This group also benefited from relatively higher use of moderate- and high-efficacy therapies, which may have contributed to more favorable outcomes. Notably, 70% of patients in this group had an EDSS score of 0 at the end of the study period—the highest proportion among all age groups.

First, the novelty of our study lies in the fact that we divided the cohort into three groups: early onset (<10 years), intermediate (10–12 years) and late-onset (after 12 years). We consider that this is a more precise division that can better highlight the characteristics of each group. Early onset with atypical onset, extended CNS lesions, a high number of relapses, and rapid disease progression; intermediate onset with an overlap between the signs and symptoms from the other two groups without fitting in any of them; and late-onset resembling the symptoms from adults. Another novelty is the holistic approach of the groups: demographic data, clinical pictures, MRI and treatment aspects, focusing on early-onset group particularities. We consider that the results of this study bring important data about MS in children and their particularities (related to clinical features, vaccination, other investigations, treatment delay and outcome, etc.) from this geographic area to complete the global picture of pediatric MS.

### 4.1. Limitations

This study has several limitations that should be considered when interpreting the results. First, the retrospective design and the relatively small number of patients in the early- and intermediate-onset groups may limit the generalizability of the findings. Second, variability in imaging protocols and MRI equipment across the study period made it difficult to consistently quantify lesion volume or count, which prevented the inclusion of detailed radiological metrics. Third, although cognitive and psychiatric symptoms were noted and discussed, the study lacked a standardized, in-depth neuropsychological assessment across all participants, which would have offered a more precise understanding of the cognitive and emotional impact of MS in children and adolescents.

### 4.2. Future Research Directions

Future research should aim to validate these findings in larger, multicenter pediatric MS cohorts with standardized imaging protocols and longitudinal monitoring. Prospective studies incorporating detailed neuropsychological testing are essential to better characterize the cognitive and emotional impact of MS in children, particularly in early-onset cases. Additionally, long-term observational and interventional studies are needed to assess the real-world efficacy of high-efficacy therapies in pediatric populations and to inform national treatment guidelines tailored to the specific needs of children and adolescents living with MS. 

## 5. Conclusions

Pediatric-onset multiple sclerosis remains a complex and incompletely understood condition, with a highly variable clinical course depending on the age at disease onset. While disease progression is often slower than in adults, early-onset cases (<10 years) tend to present with atypical symptoms, more frequent relapses, and earlier accumulation of motor and cognitive deficits.

Prompt diagnosis and timely initiation of disease-modifying therapy—particularly high-efficacy agents—may significantly improve long-term outcomes, even in settings where access to such therapies is limited. Our findings also highlight the unique profile of the intermediate-onset group (ages 10–12), which combines features from both early- and late-onset forms and may represent a transitional phenotype that warrants further investigation.

Larger prospective studies are urgently needed to better define the clinical spectrum of pediatric MS, optimize treatment strategies, and ensure equitable access to effective therapies for all affected children.

## Figures and Tables

**Table 1:** Demographic and clinical characteristics of patients.

Item	Group 1 (<10 year)	Group 2 (10–12 year)	Group 3 (>12 year)
**Gender (female:male ratio)**	1:2	1:1	2:1
**Age of onset**	11	10	99
**Age of MS diagnosis**	5	8	107
**Duration onset-dg (months)**			
<6 mo	4	4	74
>6 mo	7	6	25
**Reasons for delay dg**			
Incomplete McDonald crit	3	4	11
Incomplete investigation	3	1	8
Atypical presentation	1	0	3
Late hospital presentation	2	2	11
**MS type**			
RRMS	9	6	62
CIS	2	4	37
**Relapses number**			
**Relapse nr before diagnosis**	1–3 p; 2–7 p; 3–1 p	1–7 p; 2–0 p; 3–3 p	1–62 p; 2–30 p; 3/4–6/1 p
**Relapse nr between diagnosis–treatment**	0–5 p; 1–2 p; 2/4–2 p	0–6 p; 1–3 p; 2–0 p	0–62 p; 1–12 p; 2/3–5/2 p
**Total nr of relapses**	1–2 p; 2/3–3/1 p; 5->10–5 p; 16–1 p	1–4 p; 2–1 p; 3->8–5 p	1–38; 2–26 p; 3–15 p; 4->6–20 p
**Duration 1st–2nd relapse (months)**			
<6 mo	2	2	30
6–12 mo	3	1	13
12–36 mo	1	1	16
>36 mo	3	2	3
**Clinical picture at onset (1st event)**			
Multifocal	4	2	33
Unilateral deficit	5	5	35
Bilateral deficit	0	0	7
Balance disturbances	2	4	17
Cranial nerve palsy	4	3	20
Sensitivity disorder	0	0	32
ON	3 (2 bilat)	1 bilat	28
ADEM/ICH-like sy	3	0	2
**Clinical features during relapses**			
Unilateral deficit	1->3 ep/4 p 5->8 ep/2 p	1->2 ep/3 p 3 ep/1 p	1 ep/19 p 2 ep/7 p
Bilateral deficit	1 ep/2 p 4 ep/1 p	0	1 ep/2 p
Balance disturbances	1->3 ep/3 p 6->8 ep/2 p	3 ep/2 p	1 ep/15 p 2->3 ep/7 p
Cranial nerve palsy	1 ep/1 p 3 ep/3 p	1->2 ep/2 p	1 ep/16 p 2->4 ep/6 p
Sensitivity disorder	1->2 ep/5 p	1 ep/2 p	1 ep/22 p 2->3 ep/11 p
ON	0	1->2 ep/2 p 4 ep/1 p	1 ep/15 p 2->3 ep/4 p
ADEM/ICH-like sy	1->3 ep/3 p	0	0
**Oligoclonal bands**			
Positive (+)	7	8	81
**Initial chronic treatment**			
IG	1	1	0
Cortisone	1	0	0
IFNb	5	3	76
Fingolimod	2	4	3
RTX	0	1	0
Dimetyl fumarate	0	0	1
**Actual chronic treatment**			
IFNb	2	3	74
Fingolimod	4	5	5
RTX	1	2	0
Dimetyl fumarate	0	0	1
**Nr of chronic treatments**			
	0–1 p; 1–6 p 2–1 p; 3–2 p	0–1 p; 1–7 p 2–1 p; 4–1 p	0–20 p;2–5 p 1–74 p
**EDSS 6mo-3 year after dg**			
0	7	8	85
1–2	2	1	9
2.5–3	2	1	2–5
>3	0	0	1
**EDSS at 1–3 year of trat**			
0	7	8	92
1–2	2	2–1	9–4
2.5–3	0	0	2
>3	1	1	2–1
**Actual/Last EDSS**			
0	6	7	81
1–2	4	2	10
2.5–3	0	0	5
>3	1	1	3
**Cognitive problems**			
Normal intellect but learning difficulties	11	10	19
Borderline	2	4	10
Mild disability	2	0	4
Moderate disability	0	0	0
Severe disability	0	0	1
Depression/anxiety	0	2	30
**MRI at the first episode**			
Brain—nr of lesions			
5–10	6	5	64
>10	4	4	23
Spine—nr of lesions			
1–3	8	10	91
4–6	1	0	0
>6	0	0	7
**MRI at 1 year with the treat**			
Brain—nr of lesions			
1–3	4–0	5–3	28–4
4–6	2–0	2–0	18–3
7–10	2–0	1–0	8–1
>10			
Spine—nr of lesions			
1–3	4–1	6–2	37–6
4–6	2–1	2–1	13–3

Legend: ep = episode; p = patient; for most of the items on the left side, the numbers on the right side represent the number of patients from each group who had that item. For items of relapses before diagnosis/relapses between diagnosis and treatment and total nr of relapses, we put the nr of relapses and the corresponding nr of patients from each group (e.g., 1–3 p = three patients had 1 relapse); for the clinical features during relapses, for each item, we put the nr of episodes and the nr of patients (e.g., 1→3 ep/4 p = four patients had between 1 and 4 episodes of unilateral deficit), for the nr of chronic treatments we put the nr of DMTs and the corresponding nr of patients (e.g., 0–1 p = one patients had no treatment); for EDSS score when we had an interval as score, we put the nr of patients for each value (e.g., EDSS score 1–2 and patients nr 9–4 means that in Group 3 nine patients had EDSS score 1 and 4 patients had EDSS score 2; for nr of brain/spine lesions on MRI when for 1–3 lesions correspond 28–4 it means that in Group 3 28 patients had 1 new lesion and 4 patients had 3 lesions.

**Table 2:** Kruskal–Wallis and Chi-squared Tests—the difference in terms of EDSS score, number of brain and spinal cord lesions and duration between the first and second episode according to age of onset of MS.

	Χ^2^	df	*p*
Current EDSS _ final monitoring period when turning 18 years old	9.346	6	0.155
duration between attacks1_attack2	22.215	12	0.035
EDSS 6 months after dg	12.122	6	0.059
EDSS 1 year after dg	14.204	8	0.077
EDSS 2 years after dg	11.270	8	0.187
EDSS 3 years after dg	14.833	8	0.062
EDSS 4 years after dg	40.441	8	<0.001
EDSS 5 years after dg	52.469	8	<0.001
EDSS 6 years after dg	37.476	6	<0.001
EDSS 7 years after dg	14.644	4	0.005
EDSS at 1st year of treatment	12.447	8	0.132
EDSS at 2nd year of treatment	6.568	8	0.584
EDSS at 3rd year of treatment	12.210	8	0.142
EDSS at 4th year of treatment	32.052	6	<0.001
EDSS at 5th year of treatment	22.556	6	<0.001
EDSS at 6th year of treatment	11.092	2	0.004
EDSS at 7th year of treatment	21.148	10	0.020
Current EDSS	13.579	10	0.193
Duration between flares under treat 1	25.468	10	0.005
no new brain lesions—first MRI	7.414	6	0.284
no new spinal cord lesions—first MRI	21.054	4	0.615
no new brain lesions—MRI at 7 year form treat	42.470	6	<0.001
no new spinal cord lesions—first MRI at 7 year form treat	42.470	4	<0.001
no new brain lesions—MRI at 6 mo after dg	7.284	8	0.506
no new spinal cord lesions—MRI at 6 mo after dg	2.722	4	0.605
no relapses until diagnosis	4.718	2	0.048
no total relapses	5.218	2	0.047

## Data Availability

The original contributions presented in this study are included in the article. Further inquiries can be directed to the corresponding author.

## Abbreviations

The following abbreviations are used in this manuscript:

CFS	Cerebral fluid spine
DMT	Disease-modifying therapy
ICH	Intracranial hypertension syndrome
MRI	Magnetic Resonance Imaging
POMS	Pediatric Multiple sclerosis
